# Autonomous maneuver decision-making method based on reinforcement learning and Monte Carlo tree search

**DOI:** 10.3389/fnbot.2022.996412

**Published:** 2022-10-25

**Authors:** Hongpeng Zhang, Huan Zhou, Yujie Wei, Changqiang Huang

**Affiliations:** Aeronautics Engineering College, Air Force Engineering University, Xi'an, China

**Keywords:** autonomous air combat, maneuver decision-making, deep reinforcement learning, Monte Carlo tree search, neural networks

## Abstract

Autonomous maneuver decision-making methods for air combat often rely on human knowledge, such as advantage functions, objective functions, or dense rewards in reinforcement learning, which limits the decision-making ability of unmanned combat aerial vehicle to the scope of human experience and result in slow progress in maneuver decision-making. Therefore, a maneuver decision-making method based on deep reinforcement learning and Monte Carlo tree search is proposed to investigate whether it is feasible for maneuver decision-making without human knowledge or advantage function. To this end, Monte Carlo tree search in continuous action space is proposed and neural networks-guided Monte Carlo tree search with self-play is utilized to improve the ability of air combat agents. It starts from random behaviors and generates samples consisting of states, actions, and results of air combat through self-play without using human knowledge. These samples are used to train the neural network, and the neural network with a greater winning rate is selected by simulations. Then, repeat the above process to gradually improve the maneuver decision-making ability. Simulations are conducted to verify the effectiveness of the proposed method, and the kinematic model of the missile is used in simulations instead of the missile engagement zone to test whether the maneuver decision-making method is effective or not. The simulation results of the fixed initial state and random initial state show that the proposed method is efficient and can meet the real-time requirement.

## Introduction

Autonomous air combat through unmanned combat aerial vehicles is the future of air combat and maneuver decision-making is the core of autonomous air combat. Therefore, it is urgent to build maneuver decision-making methods. Maneuver decision-making means that the aircraft chooses the appropriate maneuver (e.g., normal overload, tangential overload, and roll angle) to change its state according to the acquired information of the target (e.g., azimuth, velocity, height, and distance), so as to defeat the target.

Air combat can be divided into within-visual-range air combat and beyond-visual-range air combat. With the development of science and technology, the detection distance of airborne radar and the range of air-to-air missiles have been increased to hundreds of kilometers. Therefore, both sides of the air combat can discover each other and launch missiles at beyond-visual-range. Besides, the process of beyond-visual-range air combat is different from that of within-visual-range air combat because the principle and operation method between radar-guided missiles and infrared (IR) missiles are different. Radar-guided missiles are supposed to be used for beyond-visual-range and IR-guided missiles for within visual range, because the detection range of the radar is longer than that of the IR detector. The IR-guided missile does not need external equipment to provide target information after it is launched. It can obtain information about the target by means of its infrared detector and then attack the target. Therefore, the aircraft can retreat after launching missiles. However, after launching, there are two stages in the attack of radar-guided missiles, which are called the midcourse guidance stage and the terminal guidance stage. In the intermediate guidance stage, the radar of the missile is not activated. Thus, it is necessary for the aircraft radar to continuously detect the target, providing the information for the missile and guiding it to the target. During the terminal phase, the missile continues to chase the target according to the information provided by its radar until it hits the target or loses the target.

Therefore, the decision-making method in within-visual-range air combat cannot be used for beyond-visual-range air combat directly, so we need to find a new decision-making method for autonomous air combat. At the same time, the existing maneuver decision-making methods rely on human knowledge, which can also be regarded as a dense reward in reinforcement learning. Thus, sparse reward means only using the result of air combat (i.e., win or not), which does not rely on human knowledge. Moreover, if the task is complex, it is difficult to define and design human knowledge or dense reward. Therefore, it is necessary to explore maneuver decision-making methods using a sparse reward.

Recently, most of the research on maneuver decision-making is focused on within-visual-range air combat (Mcgrew et al., 2010; Guo et al., 2017; Du et al., 2018; Huang et al., 2018; Li et al., 2019). You et al. (2019) proposed a constrained parameter evolutionary learning algorithm for Bayesian network parameters learning with scarce data, which can be applied to unmanned aerial vehicle autonomous mission decision-making. Wu et al. (2011) proposed the situation assessment method of beyond-visual-range air combat based on missile attack area, and introduced a new angle advantage function, speed advantage function, and height advantage function into the situation assessment model. Li et al. (2020) proposed a cooperative occupation method for autonomous air combat of multiple UAVs based on weapon attack area. They used the weapon attack area and air combat geometric description for one-to-one air combat situation assessment and established a multiple UAVs cooperative occupation model based on the encircling advantage function. Therefore, the cooperative occupation problem was transformed into a mixed integer non-linear programming problem and solved by an improved discrete particle swarm optimization algorithm. However, the flight model in this study is two-dimensional, that is, the height of both sides of air combat is always the same in air combat, and the control quantities do not include roll angle, so this study can be further improved. Wei et al. (2015) proposed a cognitive control model with three-layer structure for multi-UAVs cooperative search according to the cognitive decision-making mode of humans performing searching behavior. The mission area is carried on cognitive match, reduction, and division based on this model and the fuzzy cluster idea. The simulation experiments indicate the great performance of the fuzzy cognitive decision-making method for cooperative search. Zhang et al. (2018) proposed a maneuver decision-making method based on the Q network and Nash equilibrium strategy, and combined the missile attack area in the reward function to improve the efficiency of reinforcement learning. However, the maneuver library of this method only contains five maneuvers, which cannot meet the needs of air combat. Hu et al. (2021) proposed to use the improved deep Q network (Mnih et al., 2015) for maneuver decisions in autonomous air combat, constructed the relative motion model, missile attack model, maneuver decision-making framework, designed the reward function for training agents, and replaced the strategy network in deep Q network with the perception situation layer and value fitting layer. This method improves the winning rate of air combat, but the maneuver library is relatively simple and difficult to meet the needs of air combat.

It is worth noting that deep reinforcement learning has achieved professional performance in video games (Watkins and Dayan, 1992; Hado et al., 2016; Matteo et al., 2017), board games such as GO (Silver et al., 2016, 2017; Schrittwieser et al., 2020), real-time strategy games such as StarCraft (Oriol et al., 2019), magnetic control of tokamak plasmas (Jonas et al., 2022), data fusion (Zhou et al., 2020b), and intention prediction of aerial targets under Uncertain and Incomplete Information (Zhou et al., 2020a). Therefore, using deep reinforcement learning to improve the level of air combat maneuver decision-making is a feasible direction. AlphaStar is a multi-agent reinforcement learning algorithm based on supervised learning. It introduces league training: three pools of agents (the main agents, the league exploiters, and the main exploiters), each initialized by supervised learning, were subsequently trained with reinforcement learning. In AlphaStar, each agent is initially trained through supervised learning on replays to imitate human actions. Concretely, it uses a dataset of 971,000 replays played on StarCraft II from the top 22% of players. Therefore, it can be concluded that two features of AlphaStar are multi-agent reinforcement learning and human knowledge. However, we mainly focus on one-on-one air combat, which means that a multi-agent algorithm is not suitable and we are supposed to use a single-agent algorithm to address this problem. Meanwhile, replays of games from top players are not difficult to obtain, but it is difficult and expensive to obtain data from human pilots, which means that we cannot use supervised learning as the first phase of AlphaStar.

Ma et al. (2020) described the cooperative occupation decision-making problem of multiple UAVs as a zero-sum matrix game problem, and proposed a solution of double oracle algorithm combined with neighborhood search. In maneuver decision-making, at first, the position to be occupied by each aircraft is determined, and then the target to be attacked by each aircraft is determined, to reduce the threat and increase the advantage. Yang et al. (2020) studied the evasive maneuver strategy of unmanned combat aircraft in BVR air combat, and the problem was solved by the hierarchical multi-objective evolutionary algorithm. In this method, the decision variables are classified according to the physical meanings and then coded independently. Four escape maneuvers are designed, including turning maneuver, vertical maneuver, horizontal maneuver, and terminal maneuver. The evolutionary algorithm is used to find approximate Pareto optimal solutions and reduce invalid solutions, thus, the efficiency of the algorithm is improved. Ma et al. (2018) built an air combat game environment and train the agent with deep Q-learning.

Eloy et al. (2020) studied the attack against static high-value targets in air combat. It analyzed the confrontation process with game theory and put forward a differential game method of air combat combined with the missile attack area (Wu and Nan, 2013; Li et al., 2015; Wang et al., 2019). In this method, the air combat process is divided into the attack stage and retreat stage, while the attacker is divided into leader and wingman. In the attack stage, the leader enters the target area and launches missiles, and the wingman flies in formation. In the retreat stage, the wingman protects the leader from the missile attack of the other party. However, the flight model of aircraft is two-dimensional rather than three-dimensional. However, the authenticity of the two-dimensional motion model is worse than that of the three-dimensional motion model, so the three-dimensional motion model should have been used. He et al. (2017) proposed a maneuver decision-making method based on Monte Carlo tree search (MCTS), and it uses MCTS to find the action with the greatest air combat advantage among the seven basic maneuvers. This method verifies the feasibility of MCTS in maneuver decision-making.

While human knowledge or dense reward can make the algorithm achieve the goal quickly, it also limits the diversity and potential of the algorithm to the scope of human experience. For example, AlphaGo with human knowledge is defeated by AlphaGo Zero without human knowledge, and AlphaZero can defeat the world champion without human knowledge and has found several joseki that human players have never found before. Meanwhile, AlphaGo with human knowledge was once defeated by the world champion Lee Sedol, but AlphaGo Zero without human knowledge has not been defeated by any human players ever since. Thus, it is a reasonable conjecture that human knowledge is not good enough for training purposes for autonomous weapon deployment, and we propose a method in this article for air combat to investigate whether it is feasible for maneuver decision-making without human knowledge.

To this end, an air combat maneuver decision-making method based on deep reinforcement learning and MCTS is proposed, which aims at investigating whether it is feasible for maneuver decision-making without human knowledge or dense reward. First, different from existing methods, this method does not use human knowledge to assist the agent in maneuvering decision-making, but only uses the outcome of air combat simulations. Second, existing methods often make maneuver decisions in discrete and finite action space (e.g., maneuver library consists of finite maneuvers), however, the proposed method is based on continuous action space, which is more reasonable than discrete action space. Third, to select actions in continuous space, we proposed the method of MCTS in continuous space which is different from MCTS of existing decision-making methods. Moreover, existing methods often use missile engagement zone in simulations, but the missile may miss the target even if the target is in the missile engagement zone, therefore, the kinematic model of the missile is used in simulations instead of missile engagement zone to test whether it can hit the target, which reflects whether the maneuver decision-making method is effective (Li, 2010; Zhang et al., 2015). Our research logic is: if it works well in simulations, we may consider investigating it in the real world and modifying it if it does not work well. However, if it does not work well even in simulations, we do not consider transferring it to the real world. Therefore, we do the first step here, that is, investigating the method in simulations to make sure it works well in simulations at least before transferring it to the real world.

The main contributions are as follows: (1) To investigate whether it is feasible for maneuver decision-making without human knowledge, we propose to use the algorithm of self-play and MCTS which learns to search actions in continuous action space. (2) We provide a method to address the problem of MCTS in continuous space since MCTS cannot be applied to continuous space directly. (3) The simulation results demonstrate that although maneuver decision-making without human knowledge cannot completely defeat that with human knowledge, it is still feasible in air combat. The rest of this paper is organized as follows: In Section Aircraft model and missile model, the motion dynamics model of aircraft and missile is established. In Section Maneuver decision-making method based on deep reinforcement learning and MCTS, the process of self-play and neural network training is described (Hinton and Salakhutdinov, 2006; Goodfellow et al., 2017), and the role of human knowledge in maneuver decision-making is interpreted. In Section Experiments and results, the training results of the neural network and the simulation results of air combat are given, and the decision-making ability of the proposed method is discussed according to the simulation results. The method in this article is summarized in Section Conclusion.

## Aircraft model and missile model

The aircraft model adopts normal overload, tangential overload, and roll angle as control parameters. To simplify the complexity of the problem, the angle of attack and the angle of side slip are regarded as zero and the ground coordinate system is treated as the inertial system, meanwhile, the effects of the rotation of the earth are overlooked. The kinematic and dynamic model is shown as follows (Williams, 1990):


(1) $$\left\{ \begin{array}{l} \dot{x}=v\cos\gamma \cos\psi \\ \dot{y}=v\cos\gamma \sin\psi \\ \dot{z}=v\sin\gamma \\ \dot{v}=\;g(n_{x}-\sin\gamma )\\ \dot{\gamma }=\frac{g}{v}(n_{z}\cos\mu -\cos\gamma )\\ \dot{\psi }=\frac{g}{v\cos\gamma }n_{z}\sin\mu \end{array}\right.$$

where *x, y*, and *z* indicate the positions of the aircraft in the inertial coordinate system; γ is the pitch angle, ψ is the yaw angle, *v* is the velocity, and *g* is the acceleration of gravity. Roll angle μ, tangential overload *n*_*x*_, and normal overload *n*_*z*_ are control parameters. The kinematic model of the missile is Wang et al. (2019):


(2) $$\left\{ \begin{array}{l} {\dot{x}}_{m}=v_{m}\cos\gamma_{m}\cos\psi_{m}\\ {\dot{y}}_{m}=v_{m}\cos\gamma_{m}\sin\psi_{m}\\ {\dot{z}}_{m}=v_{m}\sin\gamma_{m} \end{array}\right.$$

where *x*_*m*_, *y*_*m*_, and *z*_*m*_ indicate the positions of the missile in the inertial coordinate system; *v*_*m*_ is the velocity, γ_*m*_ is the pitch angle, and ψ_*m*_ is the yaw angle. The dynamic model of the missile is:


(3) $$\left\{ \begin{array}{l} {\dot{v}}_{m}=\frac{(P_{m}-Q_{m})g}{G_{m}}-g\sin\gamma_{m}\\ {\dot{\psi }}_{m}=\frac{n_{mc}g}{v_{m}\cos\gamma_{m}}\\ {\dot{\gamma }}_{m}=\frac{n_{mh}g}{v_{m}}-\frac{g\cos\gamma_{m}}{v_{m}} \end{array}\right.$$

where *P*_*m*_ and *Q*_*m*_ are thrust and air resistance, *G*_*m*_ is the mass of the missile, and *n*_*mc*_ and *n*_*mh*_ are control overload in the yaw direction and pitch direction. *P*_*m*_, *Q*_*m*_, and *G*_*m*_ can be calculated by the following formula (Fang et al., 2019):


(4) $$P_{m}=\;\left\{ \begin{array}{l} 12000\;t\leq t_{w}\\ \;0\;t>t_{w} \end{array}\right.$$

(5) $$Q_{m}=\frac{1}{2}\rho v_{m}^{2}S_{m}C_{Dm}$$

(6) $$G_{m}=\;\left\{ \begin{array}{l} 173.6-8.2t\;t\leq t_{w}\\ \text{ 108 }t\text{ > }t_{w} \end{array}\right.$$

where *t*_*w*_ = 8.0s, ρ = 0.607, *S*_*m*_ = 0.0324, and *C*_*Dm*_ = 0.9. It is assumed that the guidance coefficient of proportional guidance law is *K* in control planes. The two overloads in yaw and pitch directions are defined as:


(7) $$\left\{ \begin{array}{l} n_{mc}=K\cdot \frac{v_{m}\cos\gamma_{t}}{g}\;[\dot{\beta }+\tan\varepsilon \tan(\varepsilon +\beta )\dot{\varepsilon }]\\ n_{mh}=\frac{v_{m}}{g}\frac{K}{\cos(\varepsilon +\beta )}\dot{\varepsilon } \end{array}\right.$$

(8) $$\left\{ \begin{array}{l} \beta =\arctan(r_{y}/r_{x}\;)\\ \varepsilon =\arctan(r_{z}/\sqrt{r_{x}^{2}+r_{y}^{2}}\;) \end{array}\right.$$

(9) $$\left\{ \begin{array}{l} \dot{\beta }=({\dot{r}}_{y}r_{x}-r_{y}{\dot{r}}_{x})/(r_{x}^{2}+r_{y}^{2})\\ \;\dot{\varepsilon }=\frac{\left(r_{x}^{2}+r_{y}^{2}){\dot{r}}_{z}-r_{z}({\dot{r}}_{x}r_{x}+{\dot{r}}_{y}r_{y}\right)}{R^{2}\sqrt{r_{x}^{2}+r_{y}^{2}}} \end{array}\right.$$

where β and ε are yaw angle and pitch angle of the line of sight, and $\dot{\beta }$ and $\dot{\varepsilon }$ are the corresponding derivatives. The line of sight vector is the distance vector $\vec{r}$, where *r*_*x*_ = *x*_*t*_−*x*_*m*_, *r*_*y*_ = *y*_*t*_−*y*_*m*_, *r*_*z*_ = *z*_*t*_−*z*_*m*_ and $R=||\vec{r}||=\sqrt{r_{x}^{2}+r_{y}^{2}+r_{z}^{2}}$.

The maximum overload of the missile is 40. When the minimum distance between the missile and the target is < 12 m, the target is regarded as a hit; when missile flight time exceeds 120 s and it still fails to hit the target, the target is regarded as missed; during the midcourse guidance stage, the target is regarded as missed when its azimuth relative to the aircraft exceeds 85°; during the final guidance stage, the target is regarded as missed when its azimuth relative to missile axis exceeds 70°.

## Maneuver decision-making method based on deep reinforcement learning and MCTS

He et al. (2017) uses MCTS to find the maneuver that makes the most air combat advantage among the seven basic maneuvers, in which human knowledge is used to define the air combat advantage. However, its action space is discrete and only contains seven basic maneuvers. In this paper, the search scope of maneuver is extended from seven basic maneuvers to continuous action space, which contains countless maneuvers theoretically, and human knowledge is not used to assist maneuver decision-making, but only the outcome of air combat simulations. The main idea of the proposed reinforcement learning algorithm is to use neural networks to generate the maneuver and value in each state and then use the neural network-guided MCTS to search the maneuver in the continuous action space. The maneuver selected by MCTS is more effective than the maneuver directly generated by the neural network. Then, repeat the above steps in the self-play to generate training samples and update the neural network with these training samples to make the neural network more closely match the improved maneuver and self-play winner. The repetition steps are stopped and the training is regarded as good enough usually when the rating of the agent (Silver et al., 2016, 2017; Schrittwieser et al., 2020) or the scores obtained by the agent (Mnih et al., 2015; Hado et al., 2016) does not increase visibly. The new network is used in the next iteration to make MCTS more powerful.

AlphaGo with human knowledge is defeated by AlphaGo Zero without human knowledge, and AlphaZero can defeat the world champion without human knowledge and has found several joseki that human players have never found before. Meanwhile, AlphaGo with human knowledge was once defeated by the world champion Lee Sedol, but AlphaGo Zero without human knowledge has not been defeated by any human players ever since. Therefore, we write “While human knowledge or dense reward can make the algorithm achieve the goal quickly, it also limit the diversity and potential of the algorithm to the scope of human experience” in the introduction, which mainly refers to the game of GO but not the autonomous weapon deployment. However, it is a reasonable conjecture that human knowledge is not good enough for the training purposes for autonomous weapon deployment, thus we propose this method for air combat to investigate whether it is feasible for maneuver decision-making without human knowledge.

Our method is inspired by and built upon AlphaGo Zero. However, AlphaGo Zero is not suitable for air combat because of continuous action space, so we modified it to make it able to handle continuous action space. Since AlphaGo with human knowledge is defeated by AlphaGo Zero without human knowledge, we want to know if the method without human knowledge is feasible in air combat or even better than the method with human knowledge; therefore, we investigate the problem in this paper. It is true that human knowledge is indeed useful, and we will study maneuver methods with human knowledge in future. On the other hand, considering the development of AlphaGo, although the AlphaStar approach used human knowledge, a new approach called AlphaStar Zero may appear just like AlphaGo Zero, which can defeat AlphaStar and the world champion in the game of StarCraft II without using any human knowledge.

### MCTS in continuous space

**Figure 1:**
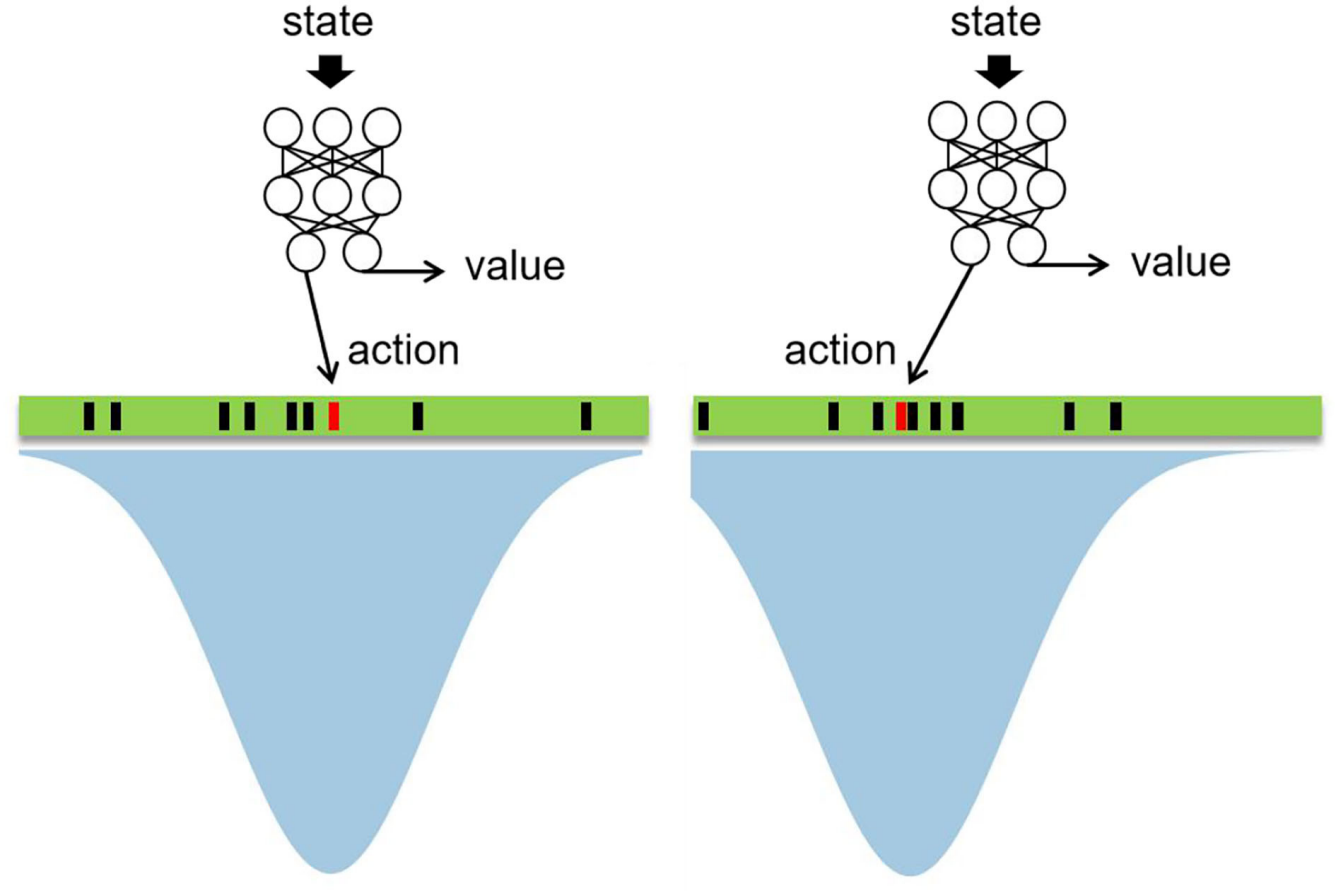
Generation and selection of action in continuous space.

MCTS is usually used for searching in discrete action space (He et al., 2017; Silver et al., 2017; Hu et al., 2021). In this paper, we use neural networks to guide MCTS as in Silver et al. (2017). Since MCTS is typically used in discrete space and cannot be used in continuous space directly, we propose the method of MCTS in continuous space to address the problem of maneuver decision-making in air combat. The generation and selection of action in continuous space are shown in Figure 1.

**Figure 2:**
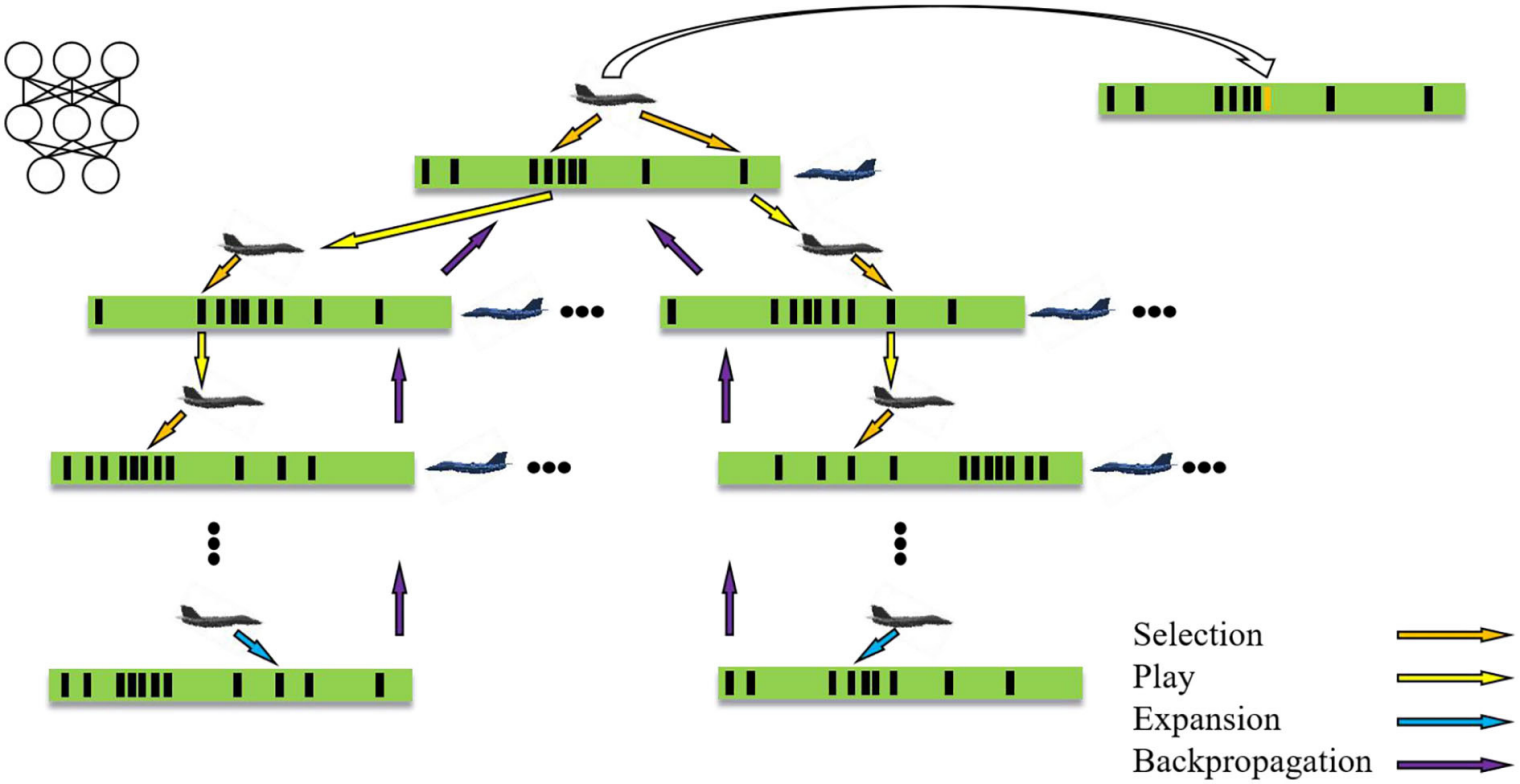
MCTS in continuous space.

The green rectangle in Figures 1, 2 is the continuous action space, which contains countless actions theoretically. Therefore, it cannot be searched by MCTS directly and we propose the following method to make MCTS able to search in continuous action space. First, a state is sent to neural networks as input and the neural network outputs the action and value according to the state, in which the action is regarded as the mean of a Gaussian distribution, the action output by the neural network is represented by the red rectangle in Figure 1. After that, a Gaussian distribution is acquired as shown in the blue shadow part in Figure 1. Then, N-1 actions are sampled from the Gaussian distribution, which are represented by the black rectangles in Figure 1, so N actions are acquired totally and MCTS is used to search for these N actions. Figure 2 illustrates the search process of MCTS in continuous space.

Each node *s* in the tree contains all actions of edges (*s, a*), and each edge stores a set {*N*(*s, a*), *W*(*s, a*), *Q*(*s, a*), *P*(*s, a*)}, where *N* represents the number of visits, *W* represents the total action value, *Q* represents the average action value, and *P* is the a priori probability of selecting this action, which can be computed by the Gaussian probability density function.

MCTS repeats four operations to find the action: selection, play, expansion, and backpropagation. Selection: take the current state as the root node, start the simulation from the root node, and stop until the simulation reaches the leaf node at time-step L. Before time-step L, the action is selected according to the a priori probability and average action value in the tree, *a*_t_ = argmax [*Q*(*s*_t_, *a*)+*U*(*s*_t_, *a*)] (Rosin, 2011),


$$U(s,a)=P(s,a)\frac{\sqrt{\sum_{b}N(s,b)}}{1+N(s,a)}$$

the probability of *a*_t_ is proportional to the maximum of *Q*(*s*_t_, *a*)+*U*(*s*_t_, *a*), in which *Q*(*s*_t_,*a*_t_) = *W*(*s*_t_,*a*_t_)/*N*(*s*_t_,*a*_t_). Here, *W*(*s*_t_,*a*_t_) is computed by the value head of neural networks, and actions are generated by the acting head of neural networks, which is different from the original MCTS used in He et al. (2017) and Hu et al. (2021) since the original MCTS chooses action randomly instead of using neural networks.

Play: when in the selection step an action is chosen, which has not been stored in the tree, the play starts. Actions are selected in self-play until the leaf node *s*_L_ is reached, and the leaf node *s*_L_ means it has not been expanded.

Expansion: the neural network is used to evaluate the leaf node *s*_L_ added to the queue, expand the leaf node *s*_L_, and each edge (*s*_L_, *a*) is initialized to {*N*(*s*_L_, *a*) = 0, *W*(*s*_L_, *a*) = 0, *Q*(*s*_L_, *a*) = 0, *P*(*s*_L_, *a*) = *p*_a_} and *p*_a_ is the priori probability of the action. This part is another different part from the original MCTS used in He et al. (2017) and Hu et al. (2021), since the original MCTS evaluates the leaf node *s*_L_ by rollouts. However, the proposed method evaluates the leaf node *s*_L_ by neural networks, that is, the MCTS is guided by neural networks.

Backpropagation: update the number of visits and value of each step t in turn, *N*(*s*_t_,*a*_t_) = *N*(*s*_t_,*a*_t_)+1, *W*(*s*_t_,*a*_t_) = *W*(*s*_t_,*a*_t_)+*v, Q*(*s*_t_,*a*_t_) = *W*(*s*_t_,*a*_t_)/*N*(*s*_t_,*a*_t_).

After several iterations, MCTS outputs the action according to *a*_t_ = argmax[*Q*(*s*_t_, *a*)+*U*(*s*_t_, *a*)] among N actions in continuous action space, as shown in the top right of Figure 2.

### Reinforcement learning from self-play

**Figure 3:**
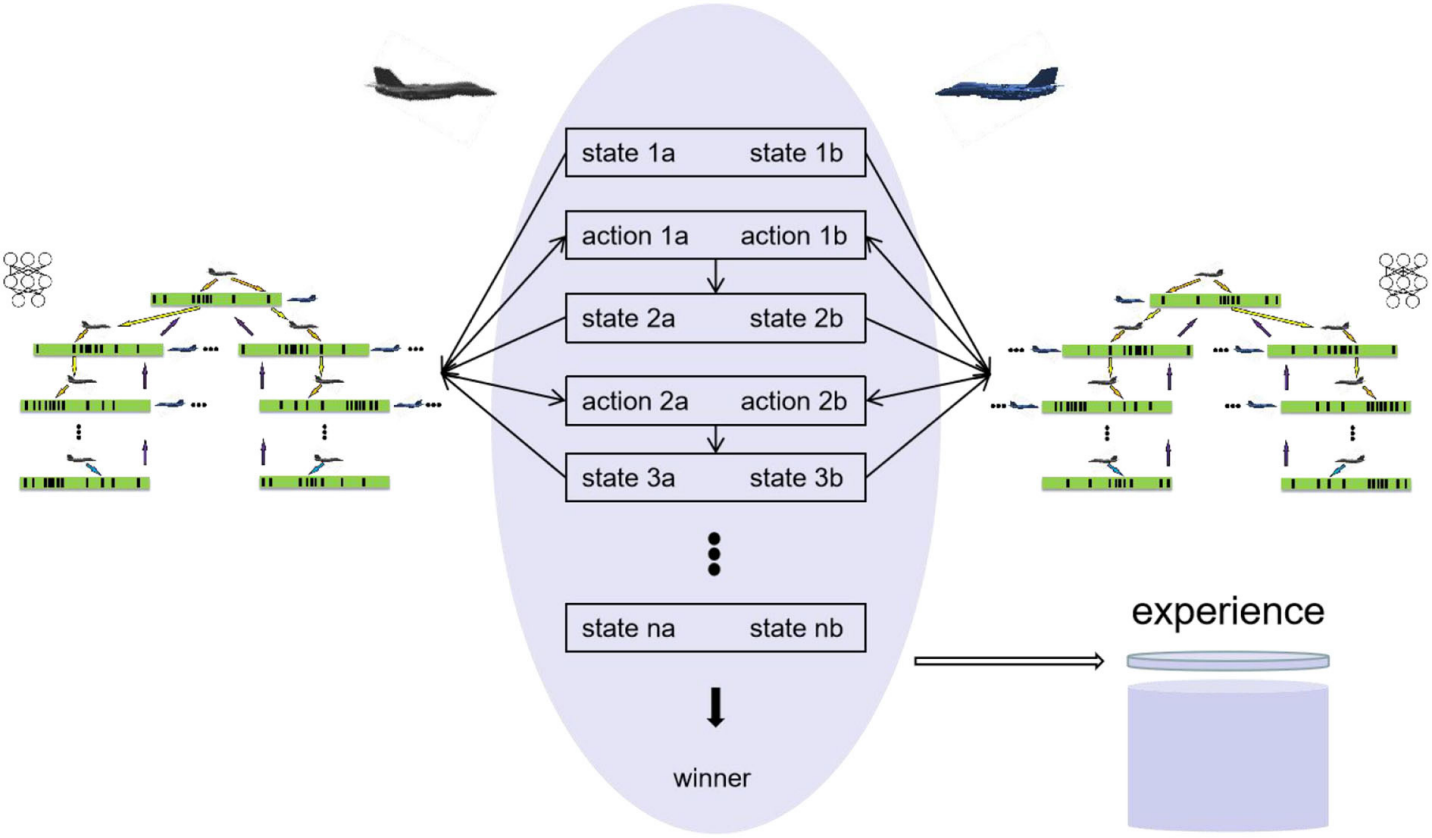
Air combat self-play.

Self-play reinforcement learning method has achieved professional performance in such games: chess (Baxter et al., 2000), othello (Sheppard, 2002), and poker (Moravcík, 2017). Therefore, this paper adopts self-play reinforcement learning for maneuver decision-making, and does not use any human knowledge. Starting from a completely random maneuver strategy, the neural network is trained by the data generated by self-play, so that the neural network can gradually produce effective maneuver strategies during the training pipeline. Figure 3 illustrates the self-play procedure.

**Figure 4:**
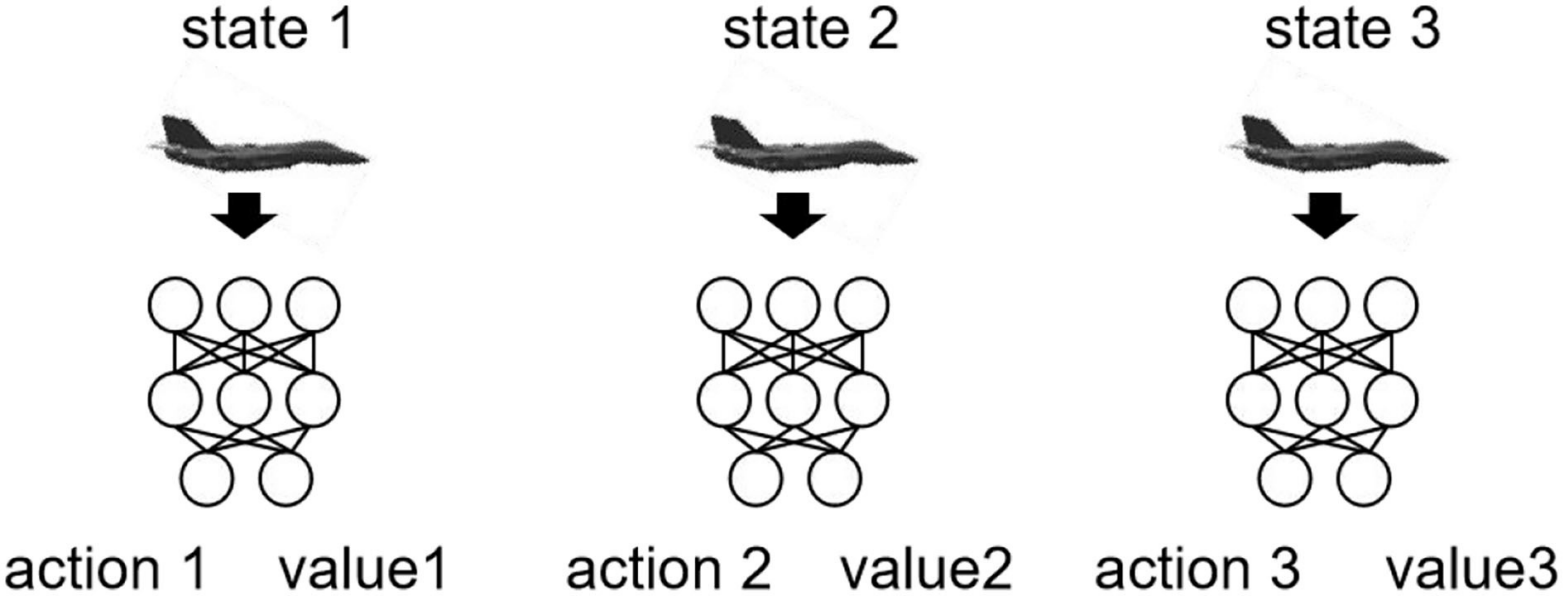
Training neural networks.

As shown in Figure 3, at each time-step, the two sides of air combat execute the maneuvers selected by MCTS and reach the next time-step and a new state. In this state, the two sides continue to execute the maneuver obtained by MCTS until the final result of the simulation is obtained. The final result at the end T is *r*_T_ = {-1, 0, 1}, where −1 represents lose, 0 represents draw, and 1 represents win. It can be seen that there is no reward function in of self-play process except the final result of air combat, that is, human knowledge is not added to self-play, which is another feature of the proposed method. Self-play uses MCTS to generate state-action pairs in each iteration and takes these state-action pairs as samples to train the neural network. As shown in Figure 3, the air combat data of each time-step t is saved as (*s*_t_, *a*_t_, *z*_t_) in the experience pool, *z*_t_ = ±*r*_T_ is the winner from the perspective of the current aircraft at time t. Uniform sampling (*s*_t_, *a*_t_, *z*_t_) from all time-steps of the last iteration of self-play is used to train the network to minimize the error of prediction value and winner and the error of neural network output and MCTS output as shown in Figure 4, and the loss function is the sum of mean square error and L2 weight regularization.

To ensure the generalization ability of the neural network, the initial state of each game is randomly selected from the following scope: azimuth scope (-45°, 45°), speed scope (250, 400 m/s), and the distance between aircraft (40, 100 km). In self-play, MCTS is used to search 90 times for each decision. The first 10 maneuvers are sampled according to the visit count of each node and the subsequent maneuvers are those with the largest visit count, so as to balance the exploration and exploitation of the algorithm.

**Figure 5:**
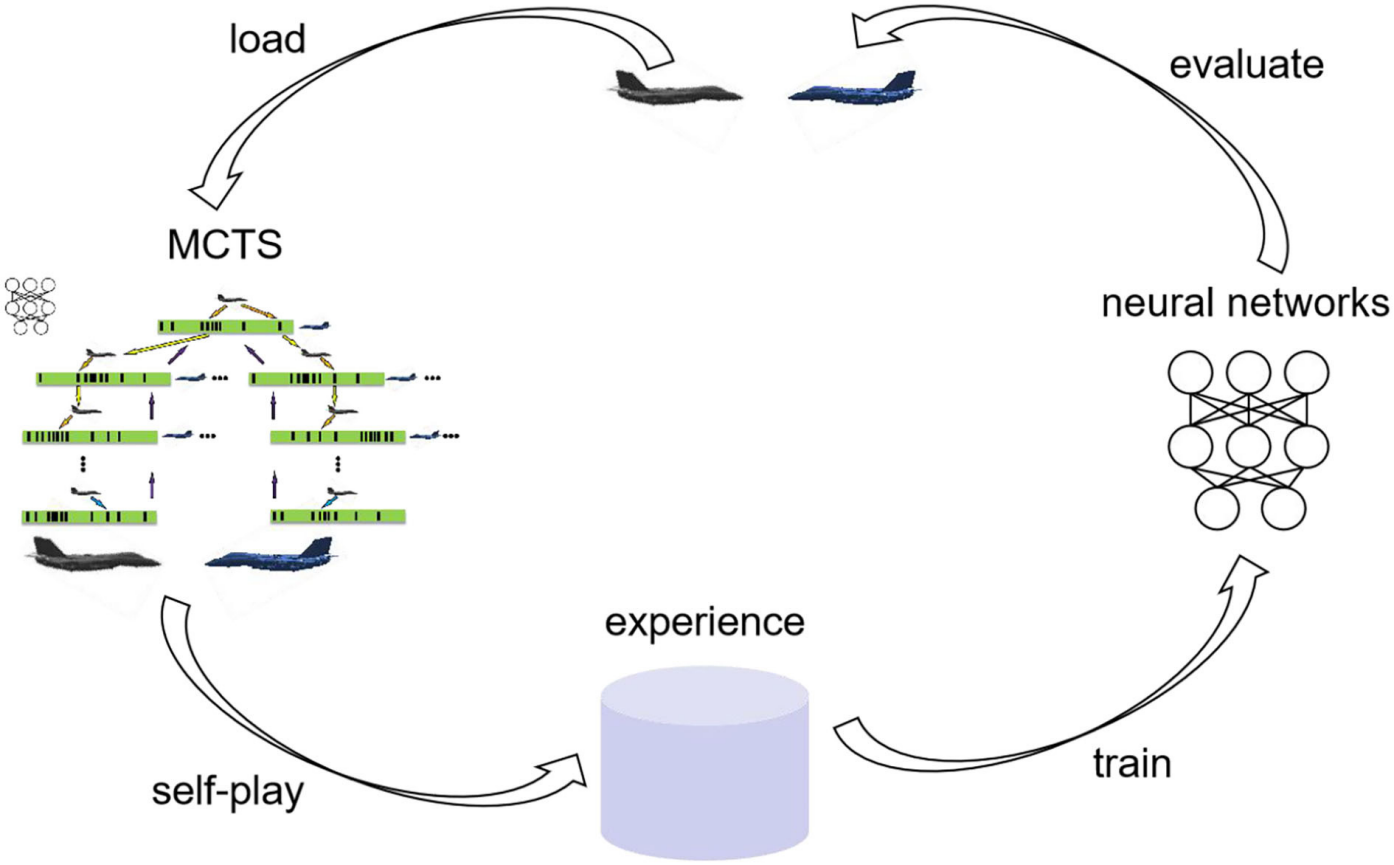
Training agent.

Figure 5 indicates the whole procedure of agent training. First, the agent generates air combat state-action pairs by MCTS in self-play and stores these data in the experience pool. Then, the neural network is trained with the data generated by 350 times of air combat self-play. During each training, 64 samples are uniformly sampled from the experience pool. The optimizer is stochastic gradient descent with a momentum of 0.9, and the L2 regularization coefficient is 0.0001. After 1,000 times of training, a new neural network is obtained and saved. To ensure the quality of the data generated from self-play, the latest neural network after each training is evaluated: use the latest neural network to simulate air combat against the current best neural network 100 times. If the number of wins of the latest network is five more than failures, the latest neural network is loaded as the current best neural network and it is used to generate data in subsequent self-play, otherwise, the latest neural network is only saved but not loaded as the current best neural network. Algorithm 1 describes one iteration of agent training in Figure 5.

**Algorithm 1:** Training agent.

Build neural networks with random weights
For iteration = 1,..., M do:
Randomly initialize state *s*_0_
For t = 0,..., max step do
State *s*_t_
Red side selects action by MCTS
Blue side selects action by MCTS
Simulate and reach the next state
If find the winner:
Beak
Else:
Continue
t = t+1
Store the state-action pairs and the winner
If required amount of experience:
Beak
Else:
Continue
Sample data from experience pool and train the neural
networks
Save and evaluate the neural networks
If wins > 5 + failures:
Load it as the current best neural network

### Air combat state and neural network architecture

The input of the neural network is a one-dimensional vector with 44 elements, which are composed of the state of the current time-step and the state of the first three time-steps. As shown in Table 1, each state contains 11 quantities: ψ, γ, *v, z, d, f*_1_, ψ_1_, γ_1_, *d*_1_, β, *f*_2_, where ψ and γ are yaw angle and pitch angle of velocity vector relative to the line of sight, *v* is the velocity of the aircraft, *z* is the flight altitude, *d* is the distance between the two sides in air combat, and *r*_1_ and *r*_2_ are the coordinates of the two sides, respectively. *f*_1_ represents whether our side launched a missile. Where ψ_1_ and γ_1_ are yaw angle and pitch angle of the missile's velocity vector relative to line of sight. *d*_1_ is the distance between the missile and the other side and *r*_m1_ is the coordinate of the missile of the side in air combat. β is heading crossing angle, that is the angle between two velocity vectors of the two sides, which is represented by *v*_1_ and *v*_2_ in Table 1. *f*_2_ represents whether the other side launched missile. The input layer is followed by three hidden layers. The number of neurons of the first two layers is 128 and the number of neurons of the third layer is 64. Finally, it output five quantities. The first three outputs are normal overload, tangential overload, and roll angle, respectively. The fourth output is whether to launch the missile and the fifth output is the value of the current state. The activation function is tanh.

**Table 1:** Air combat state.

**State**	**Symbol**	**Formula**
Yaw angle	ψ	$\psi =\psi +\int \frac{g}{v\cos\gamma }n_{z}\sin\mu \text{ dt}$
Pitch angle	γ	$\gamma =\gamma_{0}+\int \frac{g}{v}\left(n_{z}\cos\mu -\cos\gamma \right)\text{ dt}$
Velocity	*v*	*v* = *v*_0_+∫*g*(*n*_*x*_−sinγ)dt
Altitude	*z*	*z* = *z*_0_+∫*v*sinγdt
Distance between the two sides	*d*	*d* = ||*r*_1_−*r*_2_||
Launch missile	*f*_1_	0 or 1
Yaw angle of missile	ψ_1_	$\psi_{m}=\psi_{m0}+\int \frac{n_{mc}g}{v_{m}\cos\gamma_{m}}\text{dt}$
Pitch angle of missile	γ_1_	$\gamma_{m}=\gamma_{m0}\text{+}\int \frac{n_{mh}g}{v_{m}}-\frac{g\cos\gamma_{m}}{v_{m}}\;\text{dt}\;$
Distance between the missile and the other side	*d*_1_	*d* = ||*r*_*m*1_−*r*_2_||
Heading crossing angle	β	$\beta =\arccos\left(\frac{v_{1}\cdot v_{2}}{\left||v_{1}||||v_{2}|\right|}\right)$
Launch missile of the other side	*f*_2_	0 or 1

## Experiments and results

### Parameter setting

The maximum flight speed is 420 m/s, and the minimum flight speed is 90 m/s; The maximum flight altitude is 20,000 m and the minimum flight altitude is 50 m; the initial roll angle is always zero; the decision interval is 1 s and the maximum simulation time is 200 s. The outcome of the air combat simulation is defined as follows: if the missile hit the target, record it as a win; either the aircraft or the missile misses the target, it is regarded as missing the target; when the flight altitude of one side is greater than the maximum altitude or less than the minimum altitude, if the other side has launched missile and does not miss the target, record it as lose, otherwise, record it as a draw; when both sides miss the target, record it as a draw. The decision interval is 1 s, because it is common in the field since previous work (Guo et al., 2017; Du et al., 2018; Huang et al., 2018) usually uses the decision interval of 1 s. Meanwhile, a shorter decision interval requires more computational sources and a longer time span is obviously irrational.

It is true that maneuver decisions are not of any use if decisions cannot be done in a reasonable time span. Here, the rate for maneuver decision of 1 per 1 s does not mean that the maneuver is static within 1 s. For example, the aircraft takes the maneuver of changing the roll angle from 0 degrees to 45 degrees within 1 s (the case of 1 maneuver per second), thus, it gradually increases its roll angle from 0 to 45 degrees, which is a dynamic process. On the other hand, increasing the roll angle from 0 to 45 degrees may be interpreted as three maneuvers as well, for example, 0–15, 15–25, and 25–45 degrees. More importantly, even if we send several different maneuvers to the real aircraft within 1 s (such as changing the roll angle from 0 to 30 degrees, then changing it from 30 to −10 degrees, and then changing it from −10 to 50 degrees), it may not be able to realize it because of the limitations of the hardware (e.g., aircraft servomechanism). On the other hand, even if the real aircraft can realize it, it is unacceptable, because it is harmful to the aircraft to change its maneuver several times within 1 s (lack of aircraft strength). Minimum reaction time of the human brain is ~0.1 s. Meanwhile, it takes much more than 0.1 s for a human to decide what to do before the reaction, namely decision-making time. Therefore, the time span of 1 s is appropriate for a real-world application.

### Results and analysis

#### Neural networks training result

**Figure 6:**
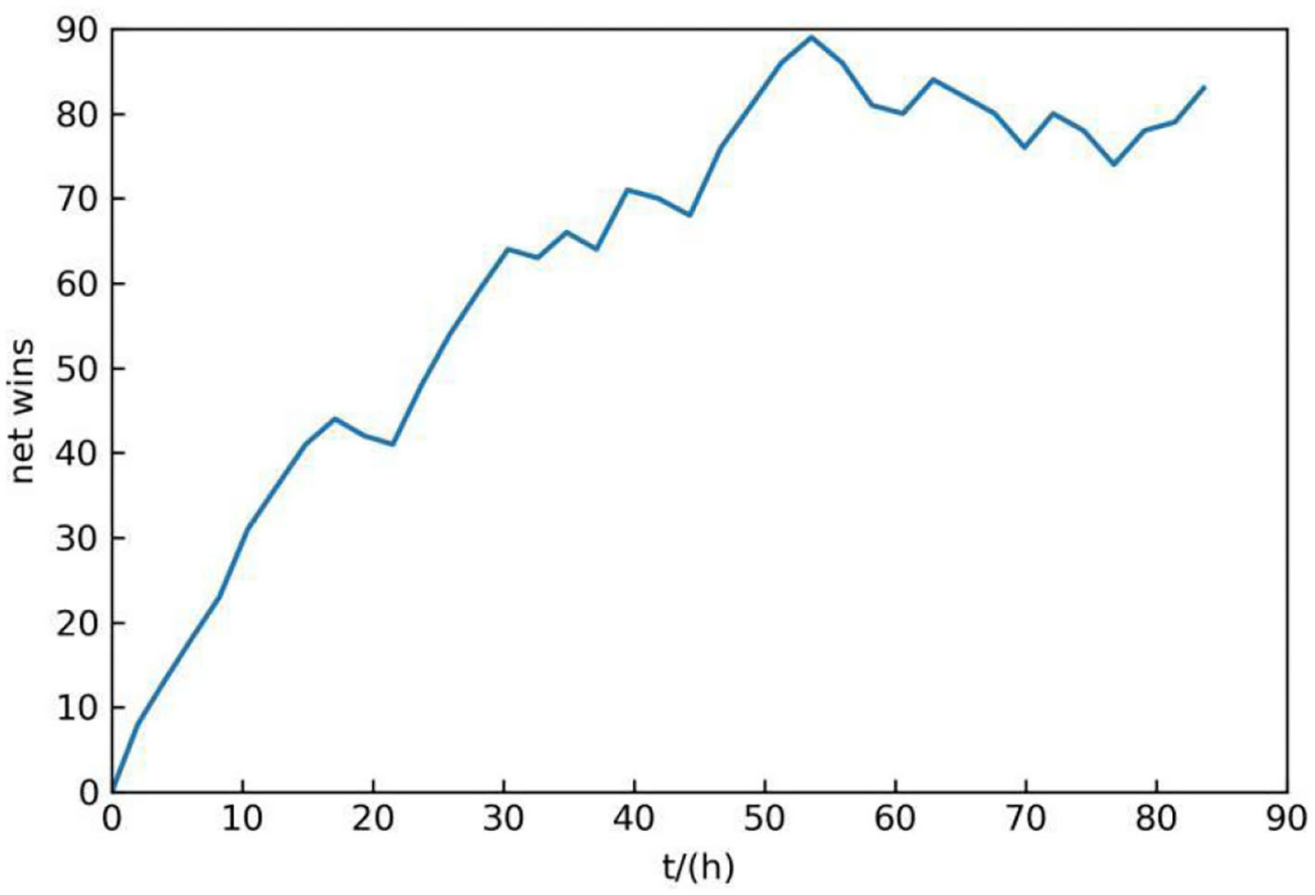
Neural networks training result.

In the process of self-play, record the net number of wins of each latest neural network in 100 times air combat, that is, subtract the number of failures from the number of wins. The reason why 100 times is selected is that 100 times is enough to distinguish the better one from both sides of the competition and does not cause too much time consumption. The total training time is about 84 h, and the change of net wins with time is shown in Figure 6.

As can be seen from Figure 6, the number of net wins is increasing along with the training. Although it sometimes decreases in the training process, it generally shows an upward trend, which indicates that the maneuvering decision-making ability of the proposed method gradually becomes more effective during self-play.

#### Air combat simulation results

We verify the effectiveness of the method we proposed by a fight against the MCTS method (He et al., 2017): (1) 100 simulations with a fixed initial state of the following simulation 2, which is a fair initial state for both sides. (2) 100 simulations with a random initial state. Table 2 indicates the win, lose, and draw times and the average time consumed by each decision-making of the proposed method. As shown in Table 2, on the one hand, the proposed method won five more times than the MCTS method, and 60 simulations is drawn. These results indicate that the proposed method is feasible and effective, even though the proposed method is just slightly better. On the other hand, when simulations started from a random initial state, the proposed method is almost the same as the MCTS method, which indicates that the initial state has a significant influence on the decision-making method. As can be seen from Table 2, for the proposed method, the average time taken for each decision-making is ~0.38 s. We also compute the average time of the original MCTS method (He et al., 2017), which is 0.11 s, this means that the proposed method is slower.

**Table 2:** Statistic results.

**Initial state**	**Win**	**Lose**	**Draw**	**Average time(s)**
Fixed	23	17	60	0.38
Random	22	21	57	0.37

**Figure 7:**
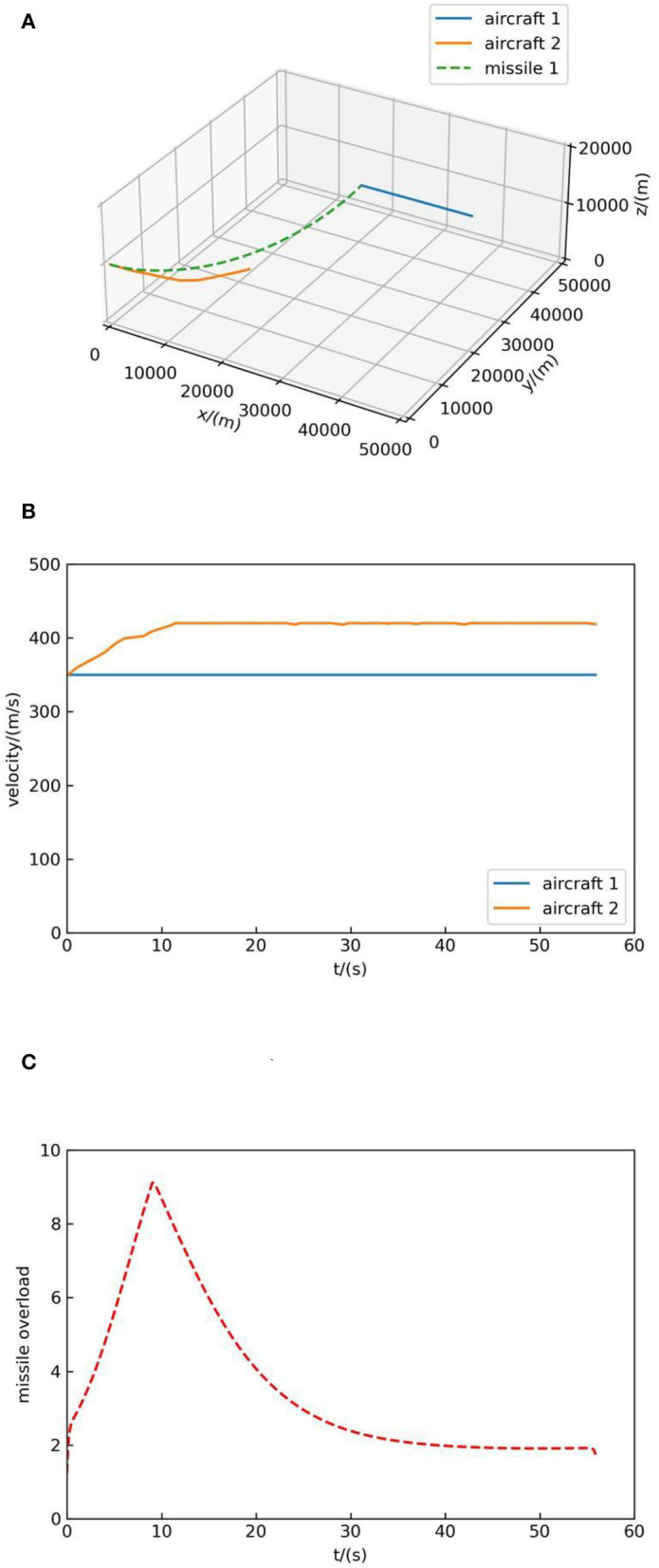
Simulation result 1. **(A)** Air combat trajectory. **(B)** Velocity. **(C)** Missile overload.

Next, we show the process of the MCTS maneuver decision-making method in continuous action space, then we show the process of the method we proposed by a fight against the MCTS method. The initial position of aircraft 1 is (40,000, 40,000, 10,000), the pitch angle is 0°, the yaw angle is 180°, and the initial velocity is 300 m/s. The initial position of aircraft 2 is (0, 0, 10,000), the pitch angle is 0°, the yaw angle is 0°, and the initial velocity is 300 m/s. Aircraft 1 moves at a constant speed in a straight line, and aircraft 2 maneuvers using the MCTS method with human knowledge. The simulation result is shown in Figure 7.

Figure 7A shows the trajectory of both sides, in which the blue solid line represents the flight trajectory of aircraft 1 and the orange solid line represents the flight trajectory of aircraft 2. In Figure 7B, the solid blue line indicates the velocity change of aircraft 1 and the orange solid line represents the velocity change of aircraft 2. Figure 7C indicates the overload change of missiles of aircraft 2. It can be seen that the MCTS method with human knowledge can react to the aircraft with simple maneuver and at the end of the simulation, the missile of aircraft 2 hit the target, which suggests the effectiveness of the MCTS method.

In simulation 2, aircraft 1 uses the proposed method and aircraft 2 uses the MCTS method (He et al., 2017), but the action space of the two methods is the same. As described in He et al. (2017), it combines the angle advantage function, distance advantage function, velocity advantage function, and height advantage function with MCTS, which means that it makes maneuver decisions with human knowledge. These advantage functions which stem from human knowledge can guide the aircraft to approach the target. However, our method uses only the final result *r*_T_ = {-1, 0, 1}, as described in Section Reinforcement learning from self-play, including no human knowledge.

**Figure 8:**
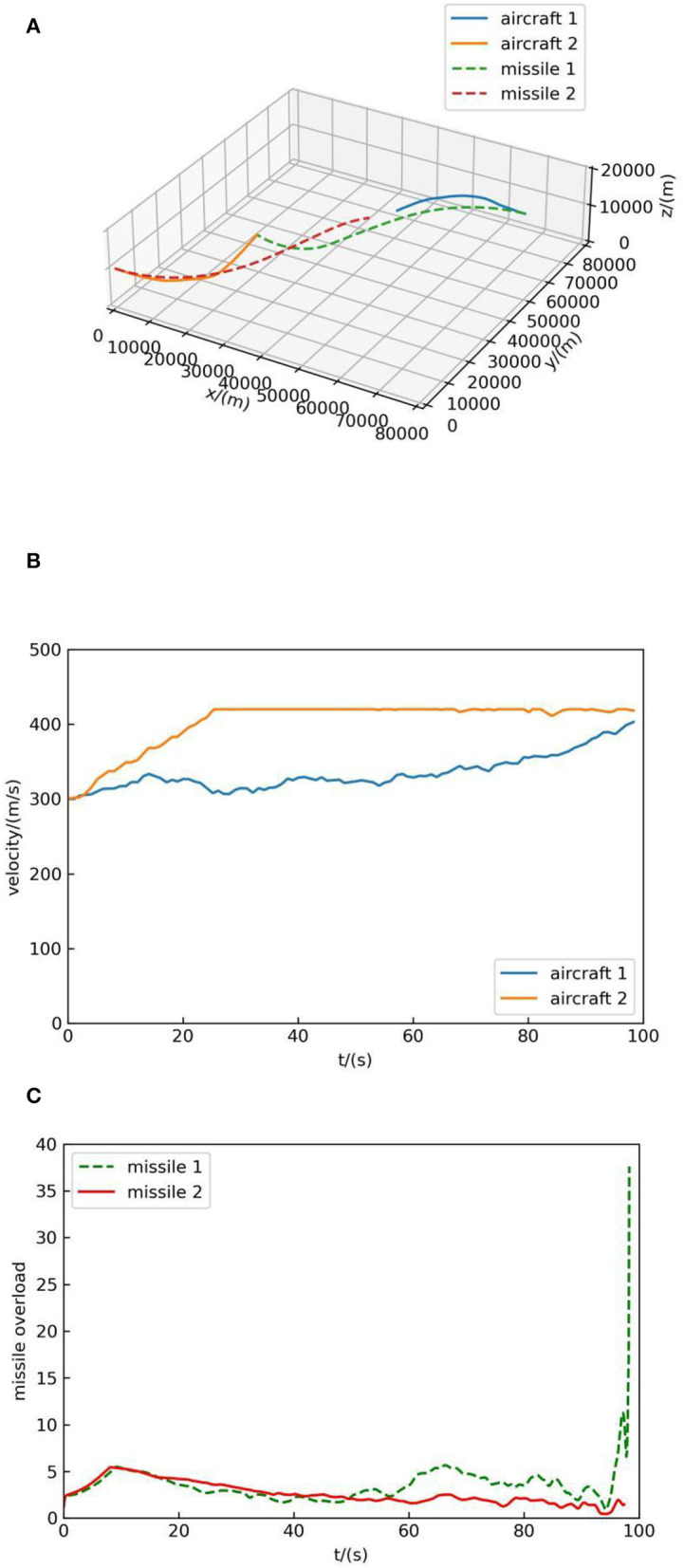
Simulation result 2. **(A)** Air combat trajectory. **(B)** Velocity. **(C)** Missile overload.

The initial position of aircraft 1 is (70,000, 70,000, 10,000), the pitch angle is 0°, the yaw angle is 180°, and the initial velocity is 300 m/s. The initial position of aircraft 2 is (0, 0, 10,000), the pitch angle is 0°, the yaw angle is 0°, and the initial velocity is 300 m/s. As can be seen that the initial situation of both sides is equal. The simulation result is shown in Figure 8.

Figure 8A shows the trajectory of both sides, in which the blue solid line represents the flight trajectory of aircraft 1, the orange solid line represents the flight trajectory of aircraft 2, the green dotted line represents the flight trajectory of missile 1, and the red dotted line represents the flight trajectory of missile 2. In Figure 8B, the solid blue line indicates the velocity change of aircraft 1 and the orange solid line represents the velocity change of aircraft 2. Figure 8C indicates the overload change of missiles of the two sides, and it can be seen from Figure 8C that the missile overload is small when it is far from the target and reaches the maximum when it hit the target.

As can be seen from Figure 8A, when the simulation begins, both sides deflect toward each other and launch missiles, but their decision-making principles are different: aircraft 1 concludes that deflecting to aircraft 2 is of high value according to a large number of self-play data, while aircraft 2 chooses to deflect to aircraft 1 because it can increase the value of the air combat advantage function. In the end, the missile of aircraft 1 hit aircraft 2, and the distance between missile 2 and aircraft 1 is about 8 km. This suggests that the proposed method without human knowledge is stronger.

**Figure 9:**
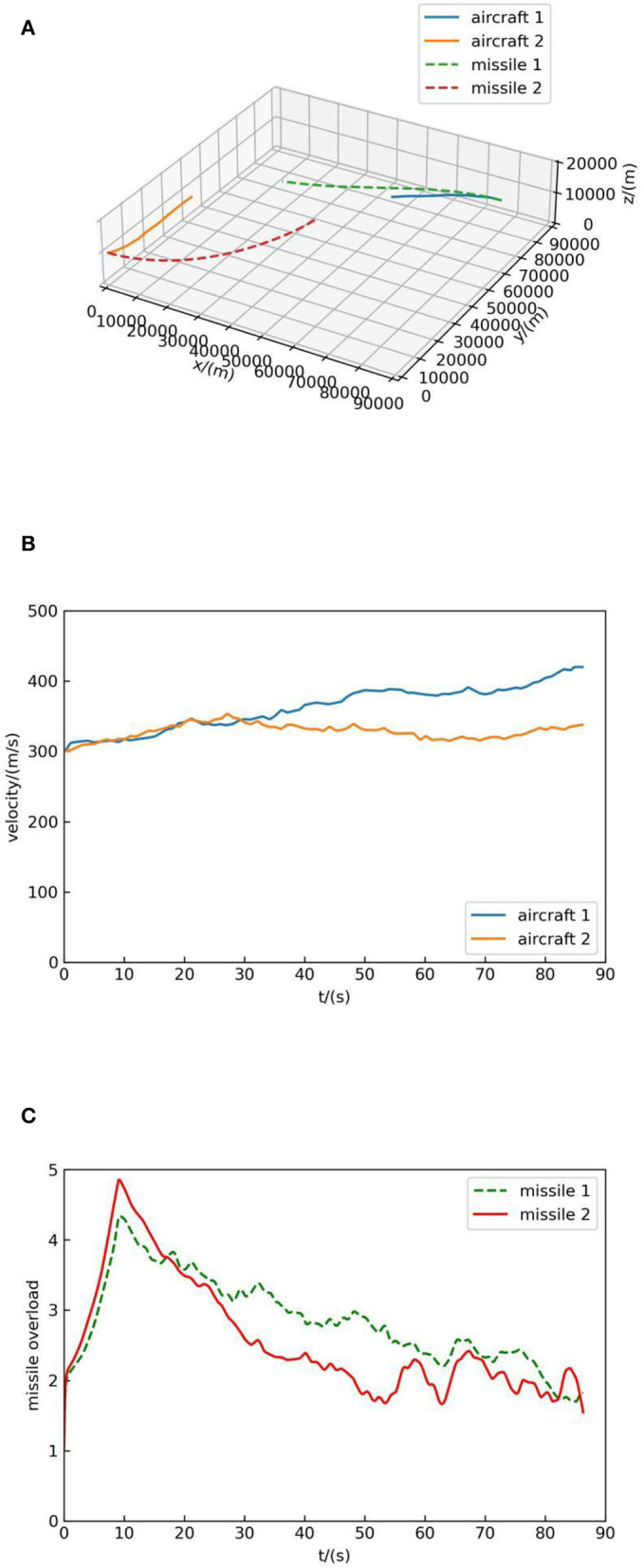
Simulation result 3. **(A)** Air combat trajectory. **(B)** Velocity. **(C)** Missile overload.

The initial position of aircraft 1 is (80 000, 80 000, 10 000), the pitch angle is 0°, the yaw angle is 180°, and the initial velocity is 300 m/s. The initial position of aircraft 2 is (0, 0, 10 000), the pitch angle is 0°, the yaw angle is 45°, and the initial velocity is 300 m/s. As can be seen that the initial situation of aircraft 2 is at an advantage. The simulation result is shown in Figure 9.

The simulation ended because the altitude of aircraft 2 exceeded the maximum altitude. The air combat advantage function of aircraft 2 includes the constraint on flight altitude to keep the altitude difference between it and the target within a certain range. However, although it used the advantage function based on human knowledge to guide maneuver decision-making, it failed to control the flight altitude properly because of the randomness of MCTS. On the contrary, the proposed method also based on MCTS can keep the flight altitude within a reasonable range without human knowledge, which indicates the effectiveness of the proposed method.

At the same time, according to Figures 7B, 8B, 9B, it can be seen that decision-making guided by human knowledge always increases the speed, while decision-making without human knowledge accelerates and decelerates, which shows that the method without human knowledge is more reasonable. Because the maximum speed is set as 420 m/s, it can be seen from the speed-increasing trend in Figures 7B, 8B, 9B that if the maximum speed is not set, the decision-making method guided by human knowledge will continue to increase the speed and always maintain the maximum speed in the subsequent air combat, which is not reasonable. Therefore, the method proposed in this paper without human knowledge is more reasonable and effective.

## Conclusion

The maneuver decision-making method based on deep reinforcement learning and Monte Carlo tree search without human knowledge is proposed in this paper. According to the simulation results, it can be concluded that a pure reinforcement learning approach without human knowledge is feasible and efficient for autonomous air combat maneuver decision-making. On the one hand, the strengths of the proposed method are as follows: (1) The method can achieve similar performance as the method with human knowledge. (2) The method is simple to implement since elaborately designed reward based on human knowledge is not necessary. (3) The method can train neural networks from scratch without using any data from human pilots, which indicates that it can be used in the domains where human data are deficient or expensive to acquire. On the other hand, the weaknesses of the proposed method are as follows: (1) The performance of the method is not as good as its counterparts in board games, such as Go and chess. (2) The time consumption of the method is more than some traditional methods. (3) It takes plenty of time for training an agent using this method.

We aim to investigate whether it is feasible for maneuver decision-making without human knowledge by means of simulations and using the results for a recommendation system or pilots in manned aircraft is out of the scope of the article. In future work, considering AlphaGo Zero without human knowledge can defeat previous algorithms and human players in Go, and it is necessary to improve the performance of the method without human knowledge since the proposed method does not completely defeat the methods with human knowledge. Meanwhile, decreasing the time consumption of the method is also another future work because the time consumption of the proposed method is more than some traditional methods. And the training procedure needs to be improved since it takes plenty of time for training an agent.

## Data availability statement

The original contributions presented in the study are included in the article/supplementary material, further inquiries can be directed to the corresponding author/s.

## Author contributions

All authors listed have made a substantial, direct, and intellectual contribution to the work and approved it for publication.

## Funding

This work was supported by the National Natural Science Foundation of China (Grant No. 62101590) and the Natural Science Foundation of Shaanxi Province (Grant No. 2020JQ-481).

## Conflict of interest

The authors declare that the research was conducted in the absence of any commercial or financial relationships that could be construed as a potential conflict of interest.

## Publisher's note

All claims expressed in this article are solely those of the authors and do not necessarily represent those of their affiliated organizations, or those of the publisher, the editors and the reviewers. Any product that may be evaluated in this article, or claim that may be made by its manufacturer, is not guaranteed or endorsed by the publisher.
